# Characterization of the Workspace and Limits of Operation of Laser Treatments for Vascular Lesions of the Lower Limbs

**DOI:** 10.3390/s22197481

**Published:** 2022-10-02

**Authors:** Bruno Oliveira, Pedro Morais, Helena R. Torres, António L. Baptista, Jaime C. Fonseca, João L. Vilaça

**Affiliations:** 1 2Ai—School of Technology, IPCA, 4750-810 Barcelos, Portugal; 2 Algoritmi Center, School of Engineering, University of Minho, 4800-058 Guimarães, Portugal; 3 LASI—Associate Laboratory of Intelligent Systems, 4800-058 Guimarães, Portugal; 4 Life and Health Sciences Research Institute (ICVS), School of Medicine, University of Minho, 4710-057 Braga, Portugal; 5 ICVS/3B’s—PT Government Associate Laboratory, 4710-057 Braga/Guimarães, Portugal

**Keywords:** laser therapy, limits of operation, medical robotics, electromagnetic tracking, vascular lesions, workspace

## Abstract

The increase of the aging population brings numerous challenges to health and aesthetic segments. Here, the use of laser therapy for dermatology is expected to increase since it allows for non-invasive and infection-free treatments. However, existing laser devices require doctors’ manually handling and visually inspecting the skin. As such, the treatment outcome is dependent on the user’s expertise, which frequently results in ineffective treatments and side effects. This study aims to determine the workspace and limits of operation of laser treatments for vascular lesions of the lower limbs. The results of this study can be used to develop a robotic-guided technology to help address the aforementioned problems. Specifically, workspace and limits of operation were studied in eight vascular laser treatments. For it, an electromagnetic tracking system was used to collect the real-time positioning of the laser during the treatments. The computed average workspace length, height, and width were 0.84 ± 0.15, 0.41 ± 0.06, and 0.78 ± 0.16 m, respectively. This corresponds to an average volume of treatment of 0.277 ± 0.093 m^3^. The average treatment time was 23.2 ± 10.2 min, with an average laser orientation of 40.6 ± 5.6 degrees. Additionally, the average velocities of 0.124 ± 0.103 m/s and 31.5 + 25.4 deg/s were measured. This knowledge characterizes the vascular laser treatment workspace and limits of operation, which may ease the understanding for future robotic system development.

## 1. Introduction

According to World Population Prospects, by 2050, one in six people in the world will be over age 65, up from one in 11 in 2019 [1]. By 2050, one in four persons living in Europe and Northern America could be aged > 65. This scenario represents a notable increase in the healthcare costs related to chronic diseases and the demand for improved and costly medical/aesthetic treatment options [2,3,4]. Due to the increased importance of looking younger and healthy and the demand for non-invasive procedures, a particular interest in laser cosmetic procedures has been registered [5]. These factors are likely to increase the number of medical spas and increase the need for aesthetic lasers, hence boosting the market growth, projected to reach EUR 6.06 billion by 2026, exhibiting a CAGR of 16.6% [6].

**Figure 1:**
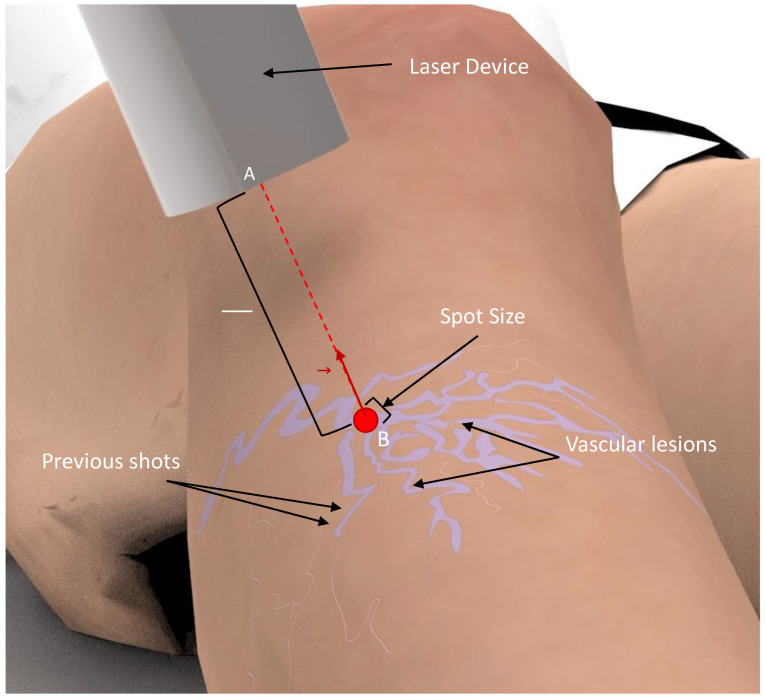
Laser treatment representation. During treatment, the laser device must be manually handled while keeping the correct distance ($\bar{AB}$) and perpendicularity ($\vec{n}$ ) to the patient’s skin. Moreover, overlapping shoots must be avoided while maximizing the treated area of the lesion. The laser light source is represented by A. The red dot and B represent the laser projection position on the patient’s leg.

To maximize treatment efficacy and minimize patient discomfort, correct laser handling and parametrization are required [7,8]. The physician needs to shoot the laser repeatedly over the vascular lesions, maintaining the laser beam perpendicular and in the center of the lesion, while keeping a distance from the target, and avoiding overlaps between subsequent pulse shots [5,7]. This process is highly repetitive and requires high precision (Figure 1). However, existing laser devices for medical and aesthetic purposes are manually handled and require a skin inspection with the naked eye [5,7]. As such, the treatment outcome is dependent on the user’s expertise, which can result in ineffective treatments and several side effects, including discoloration, hypopigmentation, and scarring [7]. Moreover, the procedure is exhausting for the physician, thereby limiting the consistency and number of consecutive treatments. These are the main limitations of the current therapy, for which there is a need for technological solutions that could make this therapy safer and more effective.

Recently, medical robotics has been proposed to improve the efficacy, precision, and safety of medical tasks with similar challenges [9]. The progress in artificial intelligence, with improved robot controlling strategies and environment perception, enabled the development of image-guided robotic solutions to execute challenging medical tasks with supervised autonomy and performance, comparable to experts [9,10,11,12]. To introduce the advantages of medical robotics for laser therapy, the development of personalized solutions to account for the task-specific requirements is fundamental [13,14,15,16]. However, the development of a robot-based system to execute a specific task is challenging and dependent on the designer’s subjectivity, specifically in medical robots. As a result, sub-optimal solutions may be developed, limiting the overall performance of the system. Indeed, technology faults are usually connected to weak task requirement specifications rather than direct device failure [16,17,18].

In this study, an analysis of the workspace and the limits of the operation of vascular laser treatment was performed. For this, laser therapy treatment sessions were monitored using a high-precision tracking system, based on electromagnetic sensors, to understand the range of movements the robot should perform and quantify the work volume of the therapy. With this information, it is expected to improve the knowledge and fully characterize the treatment in order to help in the development of a robotic-guided technology for laser treatments [19,20]. These aim to reduce the subjectivity involved in the process by empowering designers with important information to address the aforementioned task-specific requirements [13,14,17]. Thus, this study introduces the following contributions:

(1) Propose a strategy to address the robotic system development that can reduce the development and integration efforts of such a system.
(2) Quantify the workspace and limits of operation of laser treatments for vascular lesions of the lower limbs, which can be useful for the development of technologies for this specific treatment.

This paper is structured as follows. Section 2 describes the materials and methods of the proposed strategy, with the results presented in Section 3. In Section 4, the obtained results are discussed. The conclusions are given in Section 5.

## 2. Materials and Methods

**Figure 2:**
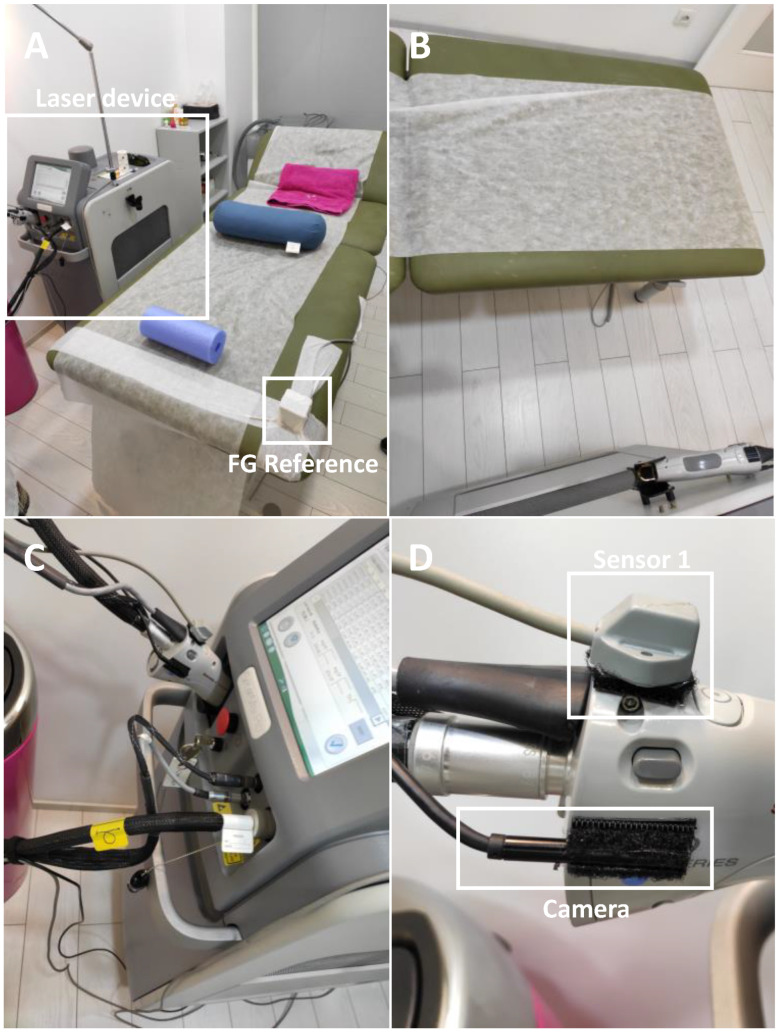
Laser treatment room. The laser device is located to the left of the surgical bed (**A**,**B**). On the handheld laser device, the electromagnetic sensor and a camera were attached (**C**,**D**). Note that FG refers to field generator.

To enable the quantification of the functional parameters and specifications required for a correct design, and the development of robotics-based solutions [13,19,20], a strategy for laser treatment monitorization was implemented. For it, the laser device was equipped with an electromagnetic system, tracking all of its 3D positions during the treatment session (Figure 2). The device used during all treatments was a candela gentledMax pro equipped with a 1064 nm Nd:YAG laser [21].

During the monitoring of the treatment, the Polhemus LIBERTY tracker was used [22]. The system allows for tracking up to 16 sensors with an update rate of 240 Hz and a latency of 3.5 ms. The device specifications report a precision of 0.8 mm on the measurements of the positions (root mean square, RMS), and a resolution of 0.0012 mm at 30 cm. The orientation measurements are stated to have a precision of 0.15° (RMS) and a resolution of 0.00040° at a distance of 30 cm. Furthermore, the system can operate up to a distance of 90 cm between the FG and the sensors, thus, it is able to be used for the monitoring of the laser treatment. The device allows for the connection of up to eight electromagnetic sensors with an I/O port using RS232 to 115,200 baud communication protocol. The system is composed of a SEU w/power supply (31 cm × 17.8 cm × 21.6 cm), a field generator (31 cm × 17.8 cm × 21.6 cm), and the electromagnetic sensors (22.9 mm × 27.9 mm × 15.2 mm).

The electromagnetic field generator (FG) was strategically positioned in the right corner of the surgical bed, in order to be close to the ROI while reducing interference during the treatment procedure (Figure 2A). Moreover, a small camera was also attached to the laser device to allow for the offline detection of the movements during the laser treatment (Figure 2C,D). Thus, the noise movements performed by the physician are neglected and the total number of positions is drastically reduced, speeding up the analysis of the data.

### 2.1. Monitoring Setup

**Figure 3:**
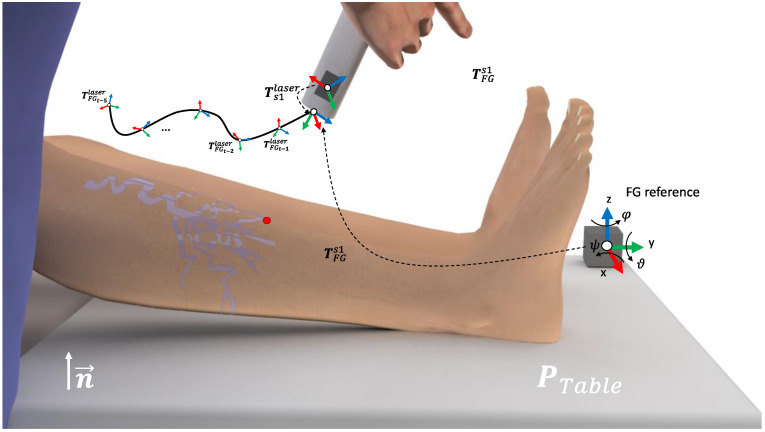
Representation of the monitoring setup of the vascular laser treatment. During the laser handling, the 3D position and orientation of the laser TCP is inferred and recorded. The red dot represents the laser projection on the patient’s leg.

Electromagnetic (EM) tracking allows for the localization of small EM sensors within a magnetic field to be emitted by a nearby EM field generator (FG). The FG works as a reference for the position and orientation measurements of the sensors. A rigid body is completely described in the space by its position and orientation in relation to a reference frame. Thus, let *R-xyz* be the orthogonal reference frame of the FG and *x*, *y*, *z* be the unit vectors of the frame axes (FG reference at Figure 3). The position of a point r’ on the rigid body, in relation to the frame *R-xyz*, is represented by $r^{\prime }={r^{\prime }}_{x}x+{r^{\prime }}_{y}y+{r^{\prime }}_{z}z$ where ${r^{\prime }}_{x}$, ${r^{\prime }}_{y}$, ${r^{\prime }}_{z}$ denote the components of the vector $r^{\prime }\in \;{\mathbb{R}}^{3}$. The frame orientation of the orthogonal frame *R’-xyz*, in relation to the frame *R-xyz*, and origin in $r^{\prime },$ are represented by the direction cosines $x^{\prime },\;y^{\prime },\;z^{\prime }$ of *R’-xyz*, in relation to *R-xyz*, defined here as a rotation matrix $R^{\prime }=\left[x^{\prime },\;y^{\prime },\;z^{\prime }\right]$. Thus, let $T^{\prime }=\left[\begin{array}{cc} R^{\prime } & r^{\prime }\\ 0^{T} & 1 \end{array}\right]$ be the matrix that represents the transformation of a vector from *R-xyz* to *R’-xyz*.

Here, a one cube-shape FG reference and one of the EM sensors for tracking the position of the laser, is denoted as s1, was used. Thus, the s1 described for the FG reference is given by $T_{FG}^{s1}$. In a common setup for the EM tracking, the FG is placed in the vicinity of the region of interest (ROI). Furthermore, let the surgical table be represented by the points $P_{Table}$, $\forall \;x\in \;P_{Table}:\;n\;\times \left(x-x0\right)=0\;\cap^{\;}x<T_{size}$, with n the orthogonal direction of the FG with the surgical table, x0 the origin of the table, in relation to the FG frame, and $T_{size}$ the size of the surgical table. Due to the impossibility of placing the sensor on the laser tip, an initial calibration was performed. First, the monitored laser point (i.e., laser tip), was placed parallel to the FG and on the pre-defined reference position x0. In this specific positioning, $T_{ref}=\left[\begin{array}{cc} I & x0\\ 0^{T} & 1 \end{array}\right]$ represents the transformation between the laser and the reference FG. Next, the rigid transformation between s1 and the laser tip can be computed as

(1) $$T_{s1}^{laser}=T_{FG}^{s1}{}^{-1}\times T_{ref}$$

Thus, during the treatment monitoring, the laser tip positioning on the treatment time *t* is given by $T_{{FG}_{t}}^{laser}=T_{{FG}_{t}}^{s1}\times T_{s1}^{laser}$(see Figure 2A).

### 2.2. Real-Time Treatment Monitoring

During the treatment session, the positioning of the laser frame at time *t* is given by $T_{FG_{t}}^{laser}$, with *t* the index of each recorded sample with time step $t_{k}$. From $T_{FG_{t}}^{laser}$, the distance and orientation between the laser tool center point (TCP) and the reference system are computed. Additionally, let ${p_{laser}}_{t}=\left[{x_{laser}}_{t},{y_{laser}}_{t},{z_{laser}}_{t}\right]$ and ${\omega_{laser}}_{t}=\left[{\varphi_{laser}}_{t},\;{\vartheta_{laser}}_{t},\;{\psi_{laser}}_{t}\right]\;$, the position and orientation in the Euler angles, are computed from $T_{FG_{t}}^{laser}$. Thus, the linear and angular handling velocities at time *t* are given by

(2) $${v_{l}}_{t}=\frac{{p_{laser}}_{t}-{p_{laser}}_{t-1}}{t_{t}-t_{t-1}}$$

and

(3) $${v_{a}}_{t}=\frac{{\omega_{laser}}_{t}-{\omega_{laser}}_{t-1}}{t_{t}-t_{t-1}},$$

respectively. To interpret the variation of the orientation of the laser beam over all of the treatment sessions, it is useful to compute a single value to represent the angle deviation $\theta_{laser_{t}}$ of the laser beam to the reference *Z*-axis (i.e., perpendicular to the surgical bed). For this, the direction vector of the laser beam at time *t* is initially computed as

(4) $${\vec{v}}_{laser_{t}}=R_{FG_{t}}^{laser}\times {\left[0,\;0,\;1\right]}^{\prime }$$

next, the angle deviation can be computed as

(5) $$\theta_{laser_{t}}=\cos^{-1}\frac{{\vec{v}}_{laser_{t}}\vec{z}\;}{\left|{\vec{v}}_{laser_{t}}\right|\times \left|\vec{z}\right|}\;$$

With $\vec{z}=\left[0,\;0,\;1\right]$ representing the direction vector of the reference *Z*-axis. Finally, the working envelope of the treatment session is defined according to the limits of the laser 3D positioning, given by the set of all lasers positions, $T_{FG}^{laser}$.

## 3. Results

The treatments were performed by a specialist in vascular laser treatments. Initially, a physical examination and a complete medical history evaluation were conducted [7,8]. During the physical examination, with the patients sitting in an upright position, the specialist assessed the type and size of the lesions. Then, only the patients suggested for laser treatment were selected, resulting in eight different laser treatments monitored during this study. Prior to starting the laser treatment, some parameters of the laser device were defined to have a maximum efficiency and minimal possibility of damage, namely wavelength, pulse duration, spot size, fluence, and cooling [5,7,8]. All of the results were later discussed and analyzed by the medical specialist to guarantee that they represent good clinical practice.

A custom software application using MATLAB (version R2021b, The Mathworks Inc., Natick, MA, USA) was developed to acquire the positions and orientations of each electromagnetic sensor and to configure the initial settings of the FGs. The data were recorded at a frame rate of 240 Hz, corresponding to the cartesian coordinates and Euler angles of each sensor to the FG reference frame, and the time stamp of the sample. For this, the I/O port using RS232 to 115,200 baud communication protocol, connected to the external computer, was used. The information was then stored in a matrix file (i.e., mat format). Following the treatments, the positioning of the laser tip was computed using the calibration strategy described in Section 2. Finally, after selecting only the positions and orientations from specific moments during the laser treatment, the trajectory map, the workspace volume, the limits of the angles, and the velocity variations (i.e., absolute and pre-position) were calculated according to the strategy described in Section 2. Note that this selection was based on the manual interpretation of the videos recorded using the camera attached at the laser device (Figure 2).

In Table 1, the quantification of the monitoring results can be analyzed. On average, the duration of the treatment sessions was 23.2 ± 10.2 min. Nevertheless, large differences were observed between the duration of the shortest and longest treatments. In respect of the limits of operation for the monitored laser treatments, an average length of 84.23 ± 15.28 cm, a height of 41.25 ± 6.15 cm, and width of 78.17 ± 15.70 cm were measured. This corresponds to a mean workspace volume of 276.3 dm^3^. The laser angle (i.e., the angle to the normal direction of the surgical table) showed a result of 40.60 ± 5.58 degrees.

**Figure 4:**
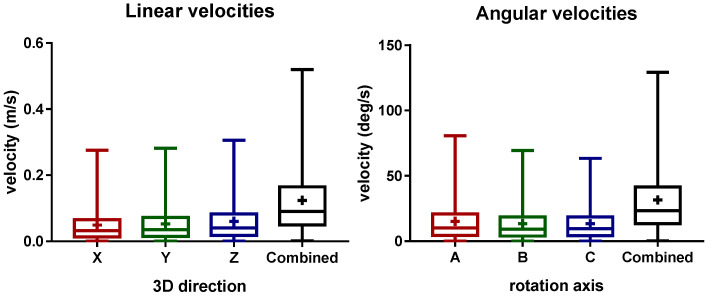
Boxplot results of the laser device velocities during the monitored treatment. The linear and rotational velocities are presented for each direction. Additionally, the combined velocity from the three components is also presented. Note that the X, Y, and Z directions represent the length, width, and height of the surgical bed, respectively.

The distribution of the linear and angular laser velocities is presented in Figure 4. Here, the mean liner velocities of 4.9 ± 5.08 cm/s, 5.288 ± 5.326 cm/s, 6.03 ± 5.895 cm/s, and 12.38 ± 10.31 cm/s were obtained for the X (length), Y (width), Z (height), and the combined directions, respectively. For the rotational velocities, the mean values of 15.22 ± 15.16 deg/s, 13.49 ± 13.19 deg/s, 13.37 ± 12.51 deg/s, and 31.5 ± 25.43 deg/s were obtained for the A (height), B (width), C (length), and the combined rotational directions, respectively. The aforementioned results show that similar velocities were measured independently of the direction. In general, during the most of the treatment, low velocities of the laser device were observed.

**Figure 5:**
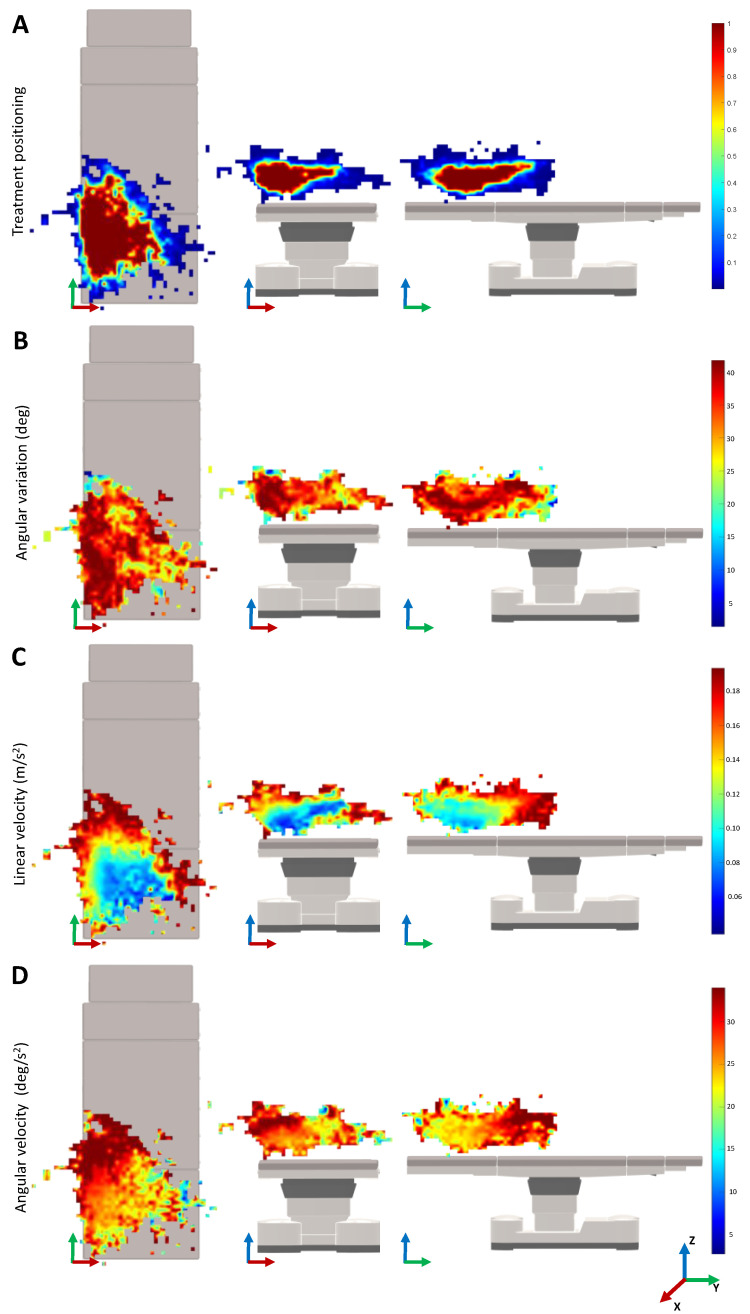
Limits of the operation when performing the vascular laser treatment task. The results for the common patient positioning are presented. The heatmaps for the occurrence of the treatment positions (**A**), median linear (**C**) and angular (**D**) velocities are represented for the top, front, and side views. The median value for the angle of the laser in respect to the Z-axis on each position is given for the same views (**B**).

Figure 5 shows the monitoring results for the movement variability depending on the 3D positioning in the treatment room. In the heatmaps of the treatment positioning, one can visualize that the most common position of the laser is on the lower left of the surgical table. The heatmap is asymmetrical, being larger over the length and width of the surgical table. Concerning the angle variation heatmaps, the positions that require a higher angle rotation of the laser beam are mainly located on the left side of the surgical table and along its length. When looking for the velocity heatmaps, the lower velocities are presented on the lower central region of the surgical table that is closer to the table on the *Z*-axis. As a last observation, the higher variations of the linear velocity of the laser device were observed when compared with the angular velocities.

## 4. Discussion

In this study, a characterization of the workspace and movements performed by the physician during the vascular laser treatment was performed. This characterization was computed from the real-time monitoring data acquired using an electromagnetic tracking setup. Due to the increase in the geriatric population and the incidence of vascular lesions on the lower limbs in adults, the number of laser treatments is expected to increase in the next few years [5,6]. Still, the current treatment is challenging and dependent on the physicians’ expertise [7]. The results of the present work allow for the identification of functional parameters for the development of a robot-based system. This information aims to reduce the subjectivity, thus, the time and costs during the development of the robotic system. Such a robotic system may reduce the dependency on experts, leading to an improvement in the treatment results. Additionally, the results of this work can be also useful for future development of other technologies related to vascular laser therapy.

Overall, the monitoring setup was able to track all of the movements during the laser treatments. Note that, an initial calibration of the monitored point (i.e., laser tip) is essential to obtain the accurate information about the treatment. In terms of time, the treatment sessions ranged from 10 to 40 min (Table 1). Note that, this time only respects the time where the laser was manually handled, the remaining interaction time between the patient and the physician was not measured. Moreover, the number of lesions to be treated with the laser may vary between patients, resulting in these time variations. The laser device was manually handled on average for 23 min per treatment, which gives an insight into how tiring this procedure can be.

Interestingly, when looking at Figure 5A, it is possible to understand that the most common position of the laser is on the left side of the table. This can be explained by the position of the laser device on that side of the room. Since the range and movements of the laser are limited by the fiber optics, by positioning the lesion to treat in that specific region, the physician has more maneuverability of the device. The physician prefers to position the patient in that region instead of moving the position of the laser device. Therefore, a possible robotic system may also be positioned on one side of the surgical bed, with the physician controlling the system on the other side.

Analyzing the heatmaps for the median linear and rotational velocities (Figure 5C,D), it can be seen that the slower velocities are located in the most visited 3D cartesian positions of the laser TCP. Therefore, the physician moves the laser slowly to achieve greater precision when shooting the laser. The higher velocities are around the most common treatment regions, which normally correspond to the movements of the physician when stopping or relocating the treatment zone. Nevertheless, as we can see in Figure 4, the velocities measured are around 12.38 ± 10.31 cm and 31.5 ± 25.43 deg/s, which can be considered low. Note that, during the treatment, it was observed that the laser shoot is not continuous. In fact, after shooting the laser a few times, the physician normally moves the laser to better analyze the patient’s skin. The patient may move, which also requires the physician to move the laser out of the treatment region. These movements are typically faster, which may lead to the increment of the overall median velocity. The integration of an electromagnetic sensor to track the patients’ movements, as well as recording the exact laser shot time, can be useful to understand these variations. This is envisioned as future work.

Remarkably, when analyzing the median angle position of the laser device during the treatment, in Table 1, a median value of 40.60 degrees was observed. This value can be explained by the positioning of the physicians’ eyes in relation to the patient’s leg. Since the physicians are normally positioned on the side of the surgical bed and looking above the patient’s leg, this orientation may allow the physician to have the most ergonomic posture to treat and analyze the patient’s skin (Figure 5B). In Table 1, it can be also seen that the width and length of the median workspace are similar. During treatment, the lesions on both the left and right legs may occur, thus, increasing the size of the workspace width. The height is relatively smaller since the patient is normally lying down on the surgical bed.

Analyzing the results from the point of view of the future development of a robot-based system for vascular laser treatment, one can conclude that the computed workspace seems to be reachable for commercially available robotic arms currently on the market [23,24,25]. The monitored treatment velocities are also not limited to the use of an already developed robotic arm. Therefore, one possible solution may rely on incorporating an available robotic arm that already fulfills specific safety and security standards, reducing the effort to develop the full system. Note that the findings of this work are based on the results of eight vascular laser treatments. Although a higher number of recorded treatments would increase the certainty of the results, this study allowed us to define the global guidelines for a robotic-based system for vascular laser treatment. In fact, the main objective is the development of the robotic system, which is already possible to begin developing, with the conclusions achieved in this study.

As for future work, it is intended to develop a robotic-based system for the guidance of vascular laser treatments, based on the results of this study. Such a system can lead to a faster, more precise, consistent, and less tiring treatment. The results of this study will aid in this process, mainly for the definition of the tool center point (TCP) and the robotic base 3D position/orientation, which are critical, since they directly affect the kinematic control of the robotic system and its 3D positioning in the task space. Furthermore, the quantification of the velocities that the robotic system must perform and the workspace of the treatment will aid in the definition of the most suitable kinematics for the system.

During this process, it is intended to use the recorded trajectories to validate the design proposals on virtual treatment scenarios (e.g., using robotic simulation systems such as coopeliaSim and Gazebo). The analysis of the device performance in a simulated clinical context may also facilitate the identification of the possible risks, thus, allowing the implementation of specific measures to mitigate or reduce certain risks before the development of the end effector tool [26,27]. Overall, the findings of this study can lead to reducing the time and costs needed for the development of the robotic system and the integration of the required safety measures of the solution.

## 5. Conclusions

This study characterizes the vascular laser treatment of the lower limbs by real-time monitoring of physicians’ movements. Overall, the computed results allowed for the visualization of the size of the laser treatment task workspace as well as the most common regions for the treatment positions. Moreover, it identified the most typical laser TCP orientation for each 3D treatment position, and the velocities during the treatment. This information is fundamental for the design of a robotic solution, to reduce the subjectivity involved in the process as well as for the implementation of safety measures (e.g., the limits of operation and safety regions). Specifically, in order to aid the development of the end effector tool and the robotic base, further validation from virtual treatment scenarios using the recorded real trajectories, is essential. The development of such a system is envisioned by the authors, in the near future.

## Figures and Tables

**Table 1:** Monitoring results of the vascular laser treatment. For each treatment session, the time of the procedure was measured. The length, height, width, volume, and laser angle (i.e., angle to the normal direction of the bed) were also computed from all of the treatment points of each monitored procedure. From this, the minimum, maximum, average, and standard deviation of the computed results are presented.

Parameters	Statistical Data			
	Min	Max	Average	Std
Time (min)	9.53	39.52	23.20	10.20
Volume (dm^3^)	136.9	409.90	276.5	93.10
Length (cm)	58.65	102.90	84.23	15.28
Height (cm)	28.49	47.51	41.25	6.15
Width (cm)	61.84	102.99	78.17	15.70
Laser angle (°)	0	111.87	40.60	5.58

## Data Availability

Not applicable.
